# Gastroenterology practice in the COVID-19 era: Ghana Association for the Study of Liver and Digestive Diseases (GASLIDD) position statement

**DOI:** 10.4314/gmj.v54i4s.16

**Published:** 2020-12

**Authors:** Yaw A Awuku, Afaa T Jibril, Ansumana S Bockarie, Amoako Duah, Kenneth Tachi, Adwoa Agyei-Nkansah, Mary Y Afihene

**Affiliations:** 1 Department of Medicine, University of Health and Allied Sciences, Ho, Ghana; 2 Department of Child Health, University of Ghana Medical School and Korle Bu Teaching Hospital, Accra, Ghana; 3 School of Medical Sciences, University of Cape Coast, Cape Coast, Ghana; 4 Department of Medicine, St. Dominic Hospital, Akwatia, Ghana; 5 Department of Medicine and Therapeutics, University of Ghana Medical School and Korle Bu Teaching Hospital, Accra, Ghana; 6 Department of Medicine, Kwame Nkrumah University of Science and Technology, Kumasi, Ghana

**Keywords:** COVID-19, GASLIDD, gastroenterology practice, practice guidance

## Abstract

The COVID-19 pandemic has impacted healthcare negatively across the globe. The practice of gastroenterology has been affected especially gastrointestinal (GI) endoscopy which is considered high risk for transmission of the virus. As a community of practitioners there is the need to share information and make evidence-based statements to guide GI practice in Ghana. This GASLIDD position statement based on the growing and rapidly evolving body of knowledge is to provide up to date information on the COVID-19 disease and guidance for the practice of gastroenterology in Ghana and beyond. It is to help the GI community of practice to maintain the highest level of health delivery and safety for our patients, staff, community and GI practitioners

**Funding:**

Self-funded

## Introduction

The pandemic caused by COVID-19 (SARS-CoV-2) emanated from the pneumonia outbreak of unknown cause in Wuhan City, Hubei Province, China, in December 2019. An infected patient may show symptom severity ranging from a mild common cold-like illness, to a severe viral pneumonia leading to acute respiratory distress syndrome that is potentially fatal. 1,2 Several strategies have been adopted to avoid exposure to the virus and these include^3^,^4^; frequent hand washing with soap and water or an alcohol-based hand sanitizer and avoiding touching the eyes, nose, and mouth with unwashed hands. Other measures such as practicing cough etiquette and general respiratory hygiene, social distancing and avoidance of crowds are important in the control of the COVID-19 pandemic.

However, the situation surrounding the COVID-19 disease continues to evolve as new evidence emerge. There is new evidence suggesting the potential transmission through droplets and perhaps faecal shedding^5^,^6^ and as such making upper and lower gastrointestinal endoscopy high risk procedures.

The Ghana Association for the Study of Liver and Digestive Diseases (GASLIDD) is a professional body made of the Physicians, Surgeons, Pathologists, Paediatricians, Radiologists, and Allied Health Professionals practicing in Ghana and abroad who are dedicated to the advancement of knowledge on the prevention, recognition, investigation and treatment of liver and digestive diseases. The association is also committed to the promotion of education and training in all fields of Gastroenterology and Hepatology and to the offering of professional/technical advice to the relevant authorities of the Ghana government and GI community of practice. As an expert group, GASLIDD provides the following information and recommendations to guide institutions and practices that provide endoscopy and gastrointestinal (GI) consultation services.

## Some Facts About COVID-19 7–13

- Asymptomatic individuals can still shed the SARSCOV-2 virus although shedding is greatest when symptoms start. (The mean incubation period is about 5 days, with a range of 0–14 days).
- Symptomatic COVID-19 adult patients commonly present with cough, fever, fatigue and sore throat.
- The elderly and individuals with co-morbidities such as heart disease, lung disease, Diabetes Mellitus, decompensated cirrhosis, HIV with low CD4 counts, and immunosuppression, (including liver and other solid organ transplant recipients) are at higher risk of developing more serious illness.
- GI symptoms such as nausea and/or diarrhea have been reported in COIVD 19 patients. There have been some reports of isolated diarrhea preceding cough and fever. Ageusia or dysgeusia are also early symptoms in the course of mild to moderate disease. Haematochezia has also been reported though rare. 14,15
- Molecular testing RT-PCR on respiratory specimens (nasopharyngeal or oropharyngeal swab or wash out) is required to confirm the diagnosis.
- The viral RNA is detectable in some GI secretions and in stool. Gastrointestinal infection and potential fecaloral transmission must be considered.
- Abnormal liver enzymes are observed in 20–30% of persons with COVID-19 infection.
- There is usually a decrease in leukocyte count with elevated white blood cell count being a poor prognostic indicator.
- Seeking medical care early for symptoms such as fever, cough, and difficult breathing, and sharing previous travel and contact history with healthcare providers improve outcome of the disease.

## Gaslidd Position on Gastrointestinal (GI) Endoscopy and Clinic Practices

- Practitioners must consider rescheduling elective endoscopic procedures. Priority can be given to procedures for cancer evaluation, prosthetic removals and evaluation of severe symptoms.
- There is an urgent need for pre-procedure screening for all patients. A special checklist (should have questions on symptoms and contacts) for screening must be administered to all patients before the decision to perform GI endoscopy or consultation is taken. Check the body temperature of patients as part of the screening process.
- Avoid bringing patients (or their escorts) who are over age 65 or have one of the recognized risks factors for severe COVID-19 disease into the medical facility.
- The GI community must acquire the requisite skills and knowledge for putting on and taking off PPEs appropriately.
- Appropriate personal protective equipment (PPE) must be worn by all members of the endoscopy team: gloves, mask, eye shield/goggles, face shields, and gown.
- Disinfection and reprocessing of endoscopes and accessories must continue along standard protocols
- There must be mandatory face mask wearing by all clients entering the premises of a health facility.
- Re-arrange seating at the clinics and procedure rooms to maintain the recommended social/physical distancing (at least 2 meters) by all patients.
- Only essential personnel should be present during procedures and at the clinics. Consider extended use or reuse of surgical masks and eye protection in accordance with hospital policies. Conservation of PPE is critical during this era.
- In the case of confirmed COVID-19 positive patients, or patients awaiting test results, isolation precautions should be taken, and re-evaluation done on the need for GI endoscopy. If considered non-urgent then procedure should be rescheduled but if considered urgent or emergency, then procedure should be performed in negative pressure room with appropriate PPEs.
- Institutions must have a dedicated phone line for follow-up for COVID-19 symptoms after consultation and procedures i.e., at day 7 and 14 post clinic visits.
- Virtual consultation systems must be encouraged to avoid overcrowding and assist with pre-hospital COVID-19 screening.
- Staff training and psychological support during this era is paramount for a safe work environment.
- Patients on immunosuppressive drugs for conditions like inflammatory bowel disease (IBD), autoimmune hepatitis, should be encouraged to continue taking their medications. The risk of disease flare outweighs the chance of contracting SARS-COV 2. These patients should also follow all the recommended guidelines for at-risk groups by avoiding crowds and limiting travel.
- Practitioners must also consider the gastrointestinal manifestations of medicines used in the treatment of COVID-19 (Hydroxychloroquine, Azithromycin, Ritonavir boosted Lopinavir, Tocilizumab and other medicines).
- Requests for liver biopsy and abdominal ultrasound should be considered carefully just like GI endoscopy. These should only be performed if considered urgent or emergency.
- There is the need for a good collaboration with the local healthcare provider or primary care physicians to limit referrals and have phone or virtual consultations with gastroenterologists at the usual referral institutions in Ghana

## Gaslidd Recommendation to the General Public

- Ensure closure of toilet cover before flushing to avoid bio-aerosols
- If possible, avoid using toilet facilities immediately after usage by another person
- Do not withhold information from health professionals to create a safe environment and improve clinical outcomes.

## Conclusion

Given the dynamic nature of COVID-19 and the overflow of evidence or publication, we will encourage the GI community of practice to refer to other sources of information. We intend to update this information as new findings and recommendations emerge. The GASLIDD position statement is to provide up to date information on the COVID-19 disease and especially the impact of this disease on the practice of gastroenterology in Ghana and beyond. It is to help the GI community of practice to maintain the highest level of health delivery and safety for our patients, staff, community and ourselves as GI practitioners.
